# Regulation of Expression and Latency in BLV and HTLV

**DOI:** 10.3390/v12101079

**Published:** 2020-09-25

**Authors:** Aneta Pluta, Juan P. Jaworski, Renée N. Douville

**Affiliations:** 1 Department of Biochemistry, National Veterinary Research Institute, 24-100 Puławy, Poland; 2 Consejo Nacional de Investigaciones Científicas y Tecnológicas (CONICET), Instituto Nacional de Tecnología Agropecuaria (INTA), Instituto de Virología, Nicolás Repetto y De los Reseros (s/n), CP1686 Hurlingham, Buenos Aires, Argentina; Jaworski.juan@inta.gob.ar; 3 Department of Biology, The University of Winnipeg, Winnipeg, MB R3B 2E9, Canada; r.douville@uwinnipeg.ca; 4 Department of Immunology, University of Manitoba, Winnipeg, MB R3E 0T5, Canada

**Keywords:** *Retrovirus*, *Deltaretrovirus*, *Human T-lymphotrophic virus type 1* (HTLV-1), *Bovine leukemia virus* (BLV), *Human immunodeficiency virus type 1* (HIV-1), long terminal repeat (LTR), viral gene regulation, transcription, latency

## Abstract

*Human T-lymphotrophic virus type 1* (HTLV-1) and *Bovine leukemia virus* (BLV) belong to the *Deltaretrovirus* genus. HTLV-1 is the etiologic agent of the highly aggressive and currently incurable cancer adult T-cell leukemia (ATL) and a neurological disease HTLV-1-associated myelopathy (HAM)/tropical spastic paraparesis (TSP). BLV causes neoplastic proliferation of B cells in cattle: enzootic bovine leucosis (EBL). Despite the severity of these conditions, infection by HTLV-1 and BLV appear in most cases clinically asymptomatic. These viruses can undergo latency in their hosts. The silencing of proviral gene expression and maintenance of latency are central for the establishment of persistent infection, as well as for pathogenesis in vivo. In this review, we will present the mechanisms that control proviral activation and retroviral latency in deltaretroviruses, in comparison with other exogenous retroviruses. The 5′ long terminal repeats (5′-LTRs) play a main role in controlling viral gene expression. While the regulation of transcription initiation is a major mechanism of silencing, we discuss topics that include (i) the epigenetic control of the provirus, (ii) the *cis*-elements present in the LTR, (iii) enhancers with cell-type specific regulatory functions, (iv) the role of virally-encoded transactivator proteins, (v) the role of repressors in transcription and silencing, (vi) the effect of hormonal signaling, (vii) implications of LTR variability on transcription and latency, and (viii) the regulatory role of non-coding RNAs. Finally, we discuss how a better understanding of these mechanisms may allow for the development of more effective treatments against *Deltaretroviruses*.

## 1. Introduction

The first reported human retrovirus and the representative of the *Deltaretrovirus* genus is *Human T-lymphotrophic virus type 1* (HTLV-1), the etiologic agent of adult T-cell leukemia (ATL) and HTLV-1-associated myelopathy/tropical spastic paraparesis (HAM/TSP) [1]. Another key member of this genus is *Bovine leukemia virus* (BLV), an oncogenic B-lymphotropic retrovirus that infects cattle and is associated with enzootic bovine leucosis caused by neoplastic proliferation of B cells [2]. HTLV-1 and BLV share a number of structural and functional similarities that include a relatively stable genome, lack of chronic viremia and low levels of viral replication [3].

Similar to other exogenous retroviruses, the deltaretrovirus genome comprises two identical copies of a single-stranded RNA molecule. Reverse transcriptase is the enzyme responsible for transcription of the genomic RNA into double-stranded proviral DNA. The integrase enzyme is essential for the insertion of virus DNA into the host chromosome, resulting in formation of a stably integrated provirus [4]. Once inserted in the host cell genome, the provirus is flanked by a 5′ and a 3′ long terminal repeats (LTRs). The provirus core encodes capsid (*gag*) and envelope proteins (*env*), as well as key enzymes (*pol*) and accessory proteins. In deltaretroviruses, accessory proteins are encoded by a sequence located 3′ of the env gene called the pX region. Both in HTLV-1 and BLV, the pX region encodes the Tax and Rex proteins that are involved respectively in transcriptional and posttranscriptional regulation of viral gene expression. Reverse genetics demonstrated that Tax and Rex are essential for viral infectivity in vivo [5,6]. In addition, other accessory genes are transcribed from the sense-strand of the pX region of BLV (R3 and G4) and HTLV-1 (p12, p13, p30). Antisense proviral transcription is also initiated from the 3′-LTR (AS1S/AS1L/AS2 in BLV and *hbz* in HTLV-1) [7,8,9,10,11]. Finally, BLV but not HTLV-1 encodes a cluster of miRNAs driven by RNAPIII. In contrast, the *Human immunodeficiency virus* (HIV) lentiviral genome codes for six accessory genes embedded within core gene regions, such as *tat*, *rev*, *vif*, *vpr*, *vpu* and *nef,* and the one putative AntiSense Protein (ASP) from the antisense [12,13,14]. HIV Tat and Rev are functional homologs of Tax and Rex proteins of HTLV and BLV that are required for viral replication [15]. The Tax proteins indirectly bind to the CRE elements located in the U3 region of the 5′-LTR. Tax activates proviral transcription through interactions with CREB/ATF family members and with histone acetyltransferases (HATs) that mediate chromatin transcription through targeted acetylation of nucleosomal histones. In contrast, HIV Tat interacts with a RNA element positioned at the 5′ end of the proviral transcript and activates cyclin T1/cdk9 complexes able to phosphorylate the C-terminal domain of RNA polymerase II [16]. Tat also associates with cellular proteins possessing HAT activity: the transcriptional co-activators p300, CREB binding protein (CBP) and the p300/CBP-associated factor (P/CAF) [17].

Retrovirus-infected cells are able to undergo a productive infection with generation of progeny virions or alternatively can enter a non-productive latent state in which viral gene expression is restricted to miRNAs and antisense transcripts [18,19]. In HTLV-1 and BLV, the amount of sense viral RNA is extremely low *in vivo.* Shortly after infection, a strong immune response controls BLV/HTLV-1 load through destruction of cells expressing viral antigens [20,21]. Only infected host cells carrying proviruses lacking viral proteins enter a non-productive latent state [22,23]. During the chronic stage of infection, deltaretroviruses promote the proliferation of infected cells through the transient action of their accessory proteins (Tax and HBZ/As2) and further propagate during mitotic division of infected cells [24,25,26]. In primary tumor cells, only very sensitive methods such as reverse transcription PCR (RT-PCR) and in situ hybridization can detect the limited amount of deltaretroviral RNA [27,28,29]. Viral reactivation can occur transiently upon exposure to exogenous stimuli (e.g., ex vivo culture, glucose and oxygen variation or receptor-mediated activation) [30]. The epigenetic state of the provirus is important for viral reactivation, and sense-strand transcription is associated with the deubiquitination of histone H2A and increase in H3K4me3 [31]. Intracellular signaling pathways activate the main regulatory elements in the 5′ LTR: Tax-response element (TRE-1, -2, -3) enhancers that interact with the cellular transcription factors CREB/ATF [32,33,34]. These complexes have been identified in cells isolated ex vivo from HTLV-1 carriers or BLV-infected sheep [34,35]. These data indicate that both viral and host cellular factors play important roles in deltaretrovirus silencing. 

Development of EBL and ATL requires Tax expression, at least in the early stages of infection. Activation of viral expression from latently-infected cells and escape from the cytotoxic lymphocyte (CTL) response is an essential step in pathogenesis. However, it still remains unclear how viral expression becomes reactivated in latent (low level-expressing) infected leukocytes in vivo. Experimentally, transcriptional activation can be initiated ex vivo by various exogenous stimuli, such as polyclonal activators, T cell receptor (TCR) ligands, antibodies (anti-CD3, anti-CD2, anti-CD28), lectins (PHA), phorbol esters (PMA), ionomycin, prostaglandins, cellular stress (oxygen deprivation, arsenic, heavy metals), histone deacetylase inhibitors (HDACi) and DNA demethylation agents [30,34,36,37,38,39,40,41]. 

**Figure 1:**
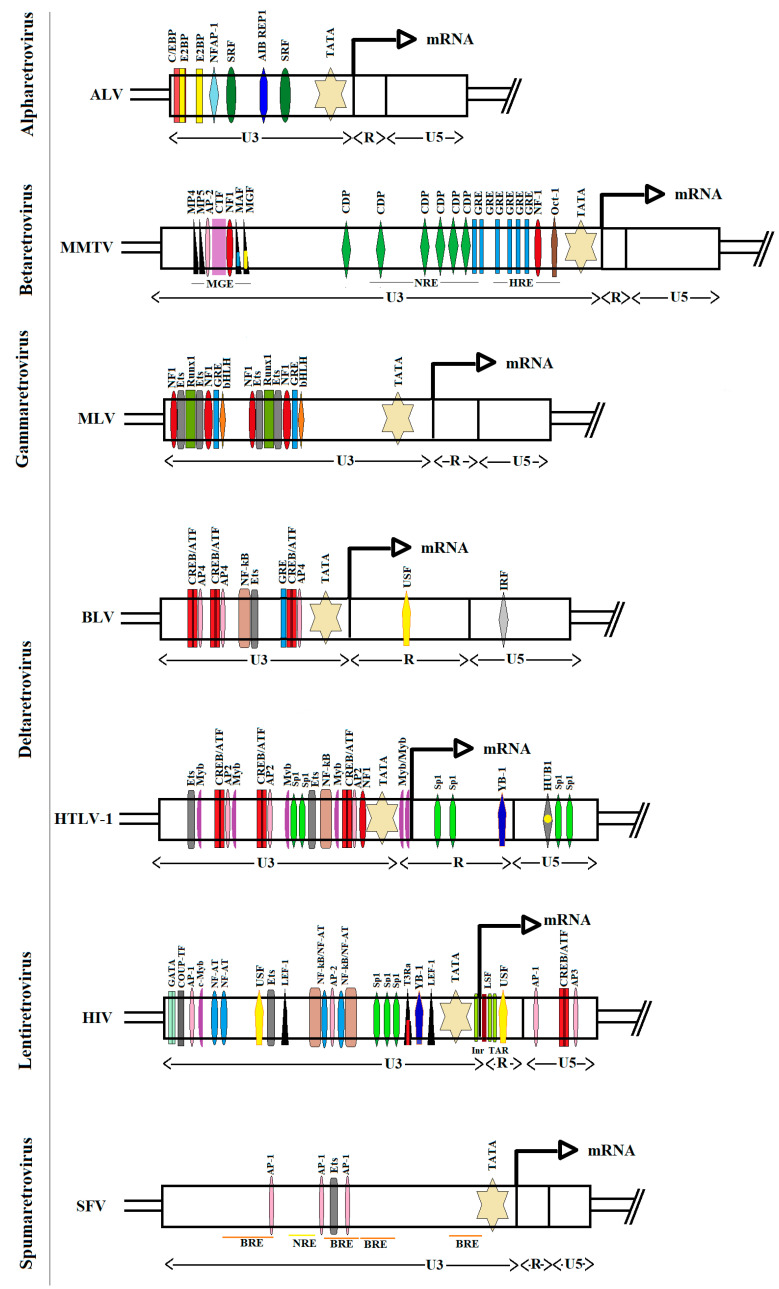
Comparison of the architecture of retroviral LTRs. Transcription factor binding sites identified within LTRs are shown with respect to the structural regions (U3, R, U5). Transcription factor binding sites are color-coded: C/EBP, CCAAT/enhancer binding protein in bright red; E2BP, E2 binding protein in light yellow; NFAP-1, nuclear factor of activated T cells (NFAT) and activator protein 1 (AP-1) binding site in sky blue; SRF, serum response factor in dark green; AIB REP1, ABA-inducible BHLH-type and Rab escort protein 1 in dark blue; MP4, 5, Myf4, 5 minimal promoter in black; AP-1, 2, 3, 4, activator protein 1, 2, 3, 4 in pink; CTF, CCAAT box-binding transcription factor in light violet; NF1, Nuclear factor 1 in dark red; MAF, mammary cell-activating factor in black-sea; MGF, mammary gland factor in black-yellow; CDP, CCAAT displacement protein in green; GRE, glucocorticoid response element in blue; Oct-1, octamer 1 in brown; Ets, E26 transformation-specific family in grey; RUNX1, runt-related transcription factor 1 in rotten-green; bHLH, basic helix-loop-helix factor in orange; CREB/ATF, cAMP-responsive element-binding protein/activating transcription factor in red-burgundy; NF-κB, nuclear factor kappa B in light brown; USF, upstream stimulatory factor in yellow; IRF, interferon regulatory factor in light gray; Myb, myeloblastosis proto-oncogene protein in violet; Sp1, specificity protein 1 in bright green; YB-1, Y-box binding protein-1 in navy blue; HUB1, HTLV-1 U5RE binding protein 1 in gray-yellow; GATA, transcriptional factor that specifically binds 5′-GATA-3′ motif in faded green; COUP-TF, chicken ovalbumin upstream promoter transcription factor in deep brown; LEF-1, lymphoid enhancer binding factor in black; T3Ra, thyroid hormone (T3) receptor alpha in black-red; LSF, late SV40 factor in olive. MMTV enhancers: MGE, mammary gland enhancer; NRE, negative regulatory element and HRE, hormone responsive element, were marked black line below LTR box. SFV elements: BRE, stands for bel-1-responsive elements and NRE, negative regulatory element, were highlighted in orange and yellow, respectively below the LTR box.

LTRs act as promoters for virus gene expression, and thus are key regulatory elements within the provirus. LTR segments are subcategorized into the U3, R, and U5 regions (Figure 1). The 5′-LTR contains transcription factor binding sites that mediate promoter activity upon regulation by a series of signaling pathways. In fact, LTR promoters contain numerous *cis*-regulatory elements, which determine the maximal rate of viral transcription initiation. However, the cell type and its differentiation state with respect to variety of cell activation signals may lead to substantial variations in transcriptional activity of a LTR depending on the chromatin context [42]. All these variables generate a remarkably wide range of levels for viral gene expression.

In this review, we summarize current knowledge on viral and host processes involved in the regulation of deltaretrovirus transcription and latency, including, (i) epigenetic control of the provirus, (ii) the cis-elements present in the LTR, (iii) enhancers with cell-type specific regulatory functions, (iv) the role virally-encoded transactivator proteins, (v) the role repressors in transcription and silencing processes, (vi) the effect of hormonal signaling, (vii) implications of LTR variability on transcription and latency, and (viii) the regulatory role of non-coding RNAs. Finally, we discuss how these integrated mechanisms contribute towards maintenance or release from viral latency in the context of antiviral therapeutics.

## 2. Epigenetic Control of Integrated Deltaretroviruses

Chromatin architecture is important for the control of viral gene expression since transcriptional activation by cellular and/or viral transactivating factors is dependent on accessibility to the promoter DNA. Epigenetic changes, such as DNA methylation and histone modifications (reviewed in [43,44]), are associated with latent retroviral infection.

### 2.1. DNA Methylation

Chromatin modifications related to transcriptional modulation are not exclusively linked to protein components; they can be associated with direct changes in the DNA. Once a retrovirus is integrated into the host genome as a provirus, it can be subject to DNA methylation as an integral part of the host cell genome. Methylation occurs mainly in sequences containing CpG dinucleotides. It has been observed that DNA is less methylated in transcriptionally active regions of the genome compared to inactive regions that are high methylated [45]. Methylation of DNA induces a change in the affinity of DNA for transcription factors, the arrangement of nucleosomes from particular regions and the interaction of histone H1 with DNA [45]. For this reason, DNA methylation of CpG islands within the 5′-LTR is associated with silencing of the integrated proviral genome. 

For HTLV-1, it was shown in vitro that DNA methylation suppresses basal, as well as Tax induced, promoter activity of the LTR [46,47,48]. The CpG methylation profile of 5′ and 3′ LTRs has been determined in persistently infected cell lines, leukemic ATL cells and PBMCs from asymptomatic HTLV-1 carriers [49]. Integrated proviruses display a selective methylation pattern characterized by hypermethylation of the 5′-LTR and hypomethylation of 3′-LTR in latently infected cell lines and ATL cells [49]. Since the sequences of the 5′ and 3′ LTRs are identical, specific mechanisms may protect 3′ LTRs from methylation. Alternatively, persistence of proviruses with this methylation pattern is favored in presence of an antiviral immune response. Selective hypermethylation of the 5′-LTR promoter downregulates transcription of the sense-strand of the viral genome. Methylation of the U3 region of 5′-LTR is associated with histone deacetylation, which disturbs the complex formation with CREB/ATF transcription factors resulting in silencing of viral transcription [50,51]. 

Initially, high levels of DNA methylation of the 5′-LTR was considered to be an essential mechanism of HTLV-1 persistence in vivo [49]. However, complete hypermethylation of the 5′-LTR in ATL cells is less frequent (14%) than partial methylation (50%) [52]. In addition, this partial methylation does not abrogate Tax-induced gene transcription in cell lines persistently infected with HTLV-1 and primary ATL cells. In these cell populations, it appears that optimal HTLV-1 expansion requires a specific level of 5′-LTR methylation that is compatible with viral expression. High *tax* gene expression is associated with unmethylated or weakly methylated 5′-LTRs at seroconversion [53]. 5′-LTR DNA methylation then accumulates in asymptomatic HTLV-1 carriers and further increases in ATL cells. ATL cells carrying highly methylated proviral DNA produce lower amounts of viral proteins, allowing escape from the host immune system [53]. 

The LTRs of HTLV-1 (i.e. HTLV-1a) and BLV (L267 cells) contain 47 and 23 CpG sites, respectively [54]. In contrast, the U3 region of HIV-1 LTR contains relatively few CpG sites (9 sites in LTR of HIV-1 HXB2 strain). There is a negative correlation between CpG methylation on HIV 5′-LTR and the residual viral load [55,56,57]. It thus appears that the role of promoter methylation in maintenance of proviral loads differs among retroviruses. In this context, it is noteworthy that HIV-1 infected patients develop viremia characterized by the presence of viral particles in peripheral blood. This is not the case in BLV and HTLV-1. 

Mechanistically, viral proteins can affect DNA methylation. In BLV, Tax downregulates DNA methyltransferase (DNMT) activity, which in turn leads to a lower level of DNA methylation in the CRE site of the promoter [54]. This mechanism facilitates interaction of CREB, CREM and ATF-1 transcription factors with their binding sites on the BLV promoter and subsequent Tax-mediated transcription [54]. In contrast, DNA hypermethylation of the BLV promoter has been associated with post-integration latency in a lymphoma-derived B-cell line [54]. As BLV mostly replicates through mitotic division of infected cells, abrogation of viral antigen expression favors escape from host’s immune response [58,59,60]. Together, these results indicate that DNA methylation in deltaretrovirus promoters is complex but clearly contributes to proviral persistence. 

### 2.2. Histone Methylation

In addition to direct DNA methylation, lysine (Lys) methylation of histones can play divergent roles in transcriptional regulation. Retroviral promoters are subject to silencing through the formation of facultative heterochromatin, which is typically created by the Polycomb repressor complex 2 (PCR2) [61]. The PRC2 complex is responsible for the di/tri methylation of Lys27 of histone H3 (H3K27me2/3) [61]. The methylation H3 at Lys9 (H3K9me) by histone methyltransferase (HMT) is also associated with gene inactivation and is a mark of constitutive heterochromatin [62]. 

Lys methylation of the N-terminal ends of histones H3 (K4, K9, K27, and K36) and H4 (K20) affect transcriptional regulation and the chromosome organization [63]. In *Saccharomyces cerevisiae,* fully activated promoters are rich in methylated H3K4, while H3K9 methylation is associated with gene silencing [63]. BLV silencing correlates with a repressed chromatin structure marked by a loss of methylation at H3K4 [64]. Concomitantly, increased H3K9 methylation is associated with transcriptional silencing even in the absence of DNA methylation, highlighting a key role of this histone modification in silencing viral genes [65]. 

In HIV, the PRC2 repressive complex modulates latency. Chromatin immunoprecipitation assays using latently HIV-infected Jurkat T cell lines showed that the catalytic subunit of the PRC2 complex, HMT Enhancer of Zeste Homolog 2 (EZH2), is present at high levels at the LTRs of silenced HIV proviruses and is rapidly displaced following proviral reactivation [61]. PRC2-mediated silencing is an important feature of HIV latency [61]. Although inhibitors of EZH2 may have therapeutic value to eradicate reservoirs of latent retroviruses, the mechanism is far more complex than expected and the strategy fails likely because of the involvement of other methyltransferases or deacetylases [66]. 

### 2.3. Histone Acetylation and Deacetylation 

Histone acetylation is among the best characterized modifications of integrated retroviral genomes (reviewed in [33]). Histone acetyltransferases (HATs) can be recruited towards proviral DNA by transcriptional factors such as nuclear factor κB (NF-κB), NFAT and C/EBPβ, where they act to modify key lysines on histones [17,43,67,68,69]. In transcriptionally active regions of the genome, these DNA-protein interactions alter chromatin architecture towards chromatin relaxation by: (i) weak interaction or absence of histone 1 (H1) with components of chromatin, and (ii) strong acetylation of histones 3 and 4 (H3 and H4) N-terminal tails. Disengagement of histone H1 and acetylation of H3 and H4 N-terminal tails prevent the formation of thick chromatin fibers by destabilizing the chromatin structure into accessible loops or hierarchical coiled structures [70]. Histone acetylation thus changes the structure and accessibility of DNA for transcriptional activators and therefore facilitates transcription by RNA polymerase II (RNAPII).

So far, most studies on histone modification of HTLV-1 proviruses were conducted using in vitro cell culture methods, where Tax expression is the primary mechanism of 5′-LTR activation through a positive feedback loop. Tax is known to interact with the chromatin remodeling complex, change nucleosome positioning, and induce transcriptional activation from the 5′-LTR [51]. Tax, together with the transcription factor complex CREB, recruits HATs such as CBP, HAT p300 and HAT P300/CBP-associated factor (PCAF) to the 5′-LTR. These interactions result in histone acetylation, nucleosomal remodeling (through BRG-1-containing SWI/SNF complexes) and chromatin relaxation, thus allowing initiation of viral transcription [33,51,71]. Furthermore, Tax directly interacts with the histone deacetylase HDAC1. Tax facilitates the dissociation of HDAC1 to the viral promoter, allowing CBP to acetylate Lys residues in H3 and H4 histone tails in the vicinity of the LTR [72]. Tax is expressed transiently in short bursts in freshly isolated HTLV-1-infected T cells and can be completely silenced in ATL, highlighting the complexity of alternate regulatory mechanisms [73,74].

HTLV-1 sense-strand transcription is associated with a significant increase in H3K4me3 and H3K9ac in HTLV-1-infected PBMC cultures [30,75]. In the ATL-derived cell line ED, H3K9 is hyperacetylated at the 3′-LTR but not at the 5′-LTR, while RNAPII is present at both LTRs [76]. This is consistent with a latency pattern where the 3′-LTR is constitutively active, but the 5′-LTR is generally suppressed [76,77]. This can be pharmacologically reversed by the HDAC inhibitor (HDACi) valproate that activates plus-strand transcription while inhibiting expression of the minus-strand [37,40,78]. In clinical trials, valproate transiently reduced the proviral loads but was inoperant in long term treatments [37,40]. Further understanding of the underlying mechanisms are required to improve the ability of HDACi to eradicate viral reservoirs [79].

These clinical trials on HTLV-1 were supported by evidence obtained in BLV. Administration of valproate to BLV-infected sheep induced BLV expression and cured leukemia [38]. The most likely hypothetical model postulates that increased virus antigen expression provokes an enhancement of anti-BLV immune response and a reduction in viral loads [38]. Mechanistically, increased H4 acetylation at the viral promoter induced by HDACi treatment is correlated with activation of BLV transcription [41,80,81]. 

## 3. LTR Regulatory Elements in Deltaretroviruses

The protein-binding signatures within retroviral LTRs are essential docking sites for initiation of transcription factor (TF) complexes. The U3 region plays a crucial role in the induction of retroviral transcription since it contains the Tax-responsive *cis*-active elements critical for the modulation of promoter activity. The cellular RNAPII initiates transcription at the U3-R boundary of the 5′ LTR. The LTR promoter contains a TATA box that provides a binding site for RNAPII, determines the site of initiation and affects the efficiency of the initiation [82]. Besides the TATA box, the basal (or core) promoter includes a CAT box and flanks upstream cis-acting elements. Being highly conserved, promoter sequences are required for adequate virus genome expression. Mutations like deletion, inversion or rearrangement of promoter sequences greatly affect virus expression [83,84]. Other cis-acting elements (enhancers) determine efficient binding of viral and cellular factors that modulate the level of transcription. 

Enhancer sequences consist of binding motifs for sequence-specific transcription factors. Notably, the complexity of cis-acting elements within the basal LTR promoters of retroviruses varies from relatively few in the case of *Avian Leucosis Virus* (ALV) to the numerous elements in case of HTLV-1/BLV group and HIV-1 retroviruses [85]. A precise understanding of this complexity is necessary to understand control of virus expression [86]. Figure 1 depicts the TF binding sites within deltaretroviruses, as compared with HIV, ALV, *Mouse Mammary Tumor Virus* (MMTV), *Murine Leukemia Virus* (MLV) and *Simian Foamy Virus* (SFV).

The main TFs involved in deltaretrovirus transcription are: cAMP-responsive element-binding protein/activating transcription factor (CREB/ATF), activating enhancer binding protein (AP), erythroblast transformation specific family members (Ets), nuclear factor kappa B (NF-κB), specificity protein (Sp) and myeloblastosis proto-oncogene protein (c-Myb). Cell type-specific TF for deltaretroviruses include: PU.1 and Spi-1 (Ets family members, which are predominantly expressed in macrophages, B and T lymphocytes), CREB and ATF1 (abundantly expressed in T- and B-cells), and c-Myb (primarily expressed in hematopoietic cells). Unlike HIV, deltaretroviruses do not contain binding sites for nuclear factor of activated T cells (NF-AT), T-cell specific/lymphoid enhancer binding factors (TCF/LEF-1) and chicken ovalbumin upstream promoter transcription factor (COUP-TF).

Enhancer sequences are usually 10-15 base pairs in length and may be located within the first ≈200 base pairs upstream of the cap site [87]. An example of an enhancer is provided by the Tax-Responsive Elements (TRE1, 2 and 3) that contain three imperfectly conserved cAMP-response elements (CREs) (Figure 1). The TRE-1 element binds CREB/ATF family members, synergizes with TRE-2, and is regulated upon recruitment of Ets, Elk-1, c-Myb and Sp1 and Sp3 [88,89,90,91]. The interplay between transcription factors in this region determines the level of viral expression [92,93]. 

As for HTLV-1, the major regulatory elements in BLV are three repeated 21-bp TREs that include imperfectly conserved CREs matching the consensus sequence 5′-TGACGTCA-3′ [94]. These CRE sites interact with cellular transcription factors ATF-1, ATF-2 and CREB [95]. The virus-encoded Tax transactivator increases the DNA binding activity of CREB/ATF proteins by interacting with their bZip domains, which positively regulates the activation of BLV transcription [96]. Besides the CRE elements, each TRE sequence includes an Ebox as a target sequence for the AP4 transcription factor [97]. Additionally, the U3 region contains several other response elements, such as: a NF-κB binding site, a glucocorticoid response element (GRE) and PU.1/Spi-B binding sites for ETS transcription factor family proteins [98,99,100]. 

Additional elements of transcription control have been identified within R and U5 sequences of 5′ LTR. For HTLV-1, the downstream regulatory element (DRE) in the R region includes the YB-1 binding site that is required for basal transcription [101]. The U5 repressive element (U5RE) contains Sp1 and HTLV-1 U5RE binding protein 1 (HUB1) binding sites, which repress viral LTR-mediated expression [91,101]. Similarly, regulatory sequences downstream of the transcription start site in BLV include: an upstream stimulatory factor (USF) binding site, downstream activator sequence (DAS) and an interferon regulatory factor (IRF) binding site, which stimulate viral gene expression [102,103,104]. Thus, regulatory elements located in R or U5 regions increase the strength of the U3 promoter-enhancer unit and provide a mechanism to broaden the viral response to stimulating factors or regulate transcription in cell-type dependent manner.

## 4. A Variety of Enhancers with Cell-Type Specific Regulatory Functions

Despite basic similarities among retroviruses, there are several distinctive features with respect to their transcriptional regulatory elements located in 5′-LTR that determine the level of viral gene expression, as well as control of cellular tropism of retrovirus replication. 

HTLV-1 gene expression is dependent on several cellular transcription factors and their different expression profiles within a given infected cell. The main cellular targets of HTLV-1 in infected individuals are CD4^+^ T lymphocytes [105,106]. HTLV-1 has also been shown to infect CD8^+^ T cells, B cells, monocytes, dendritic cells and microglial cells in vivo, although the role of these cell populations in latency and infection remains poorly understood [107,108,109]. HTLV-1 sense-strand transcription depends on the expression profiles of cellular transcription factors present in these infected cells. After integration into the host cellular genome, the HTLV-1 provirus is transcriptionally regulated by ubiquitously expressed Sp1 [110]. The high-affinity Sp1 binding site in the HTLV-1 promoter contributes to Tax-independent basal expression of the virus. Yet, Sp1 only modestly activates HTLV-1 transcription when examined both in vivo and in vitro [110]. Once Tax is expressed, it strongly transactivates viral gene expression via CREB/ATF and the pleiotropic coactivators CBP and HAT p300 [111]. HTLV-1 infection of T lymphocytes also leads to an increase in intracellular levels of cAMP, which further activates the CREB/ATF factors family through protein kinase A (PKA)-mediated phosphorylation [112]. Phospho-CREB recruits CBP and leads to increased promoter activation [112]. Elevated cAMP levels in HTLV-carrying cells during latency and transformation may maintain basal transcription from the viral LTR at low levels even without Tax expression [113]. Alternatively, cAMP might be important for maintenance phosphorylation of CREB and other protein kinase A (PKA)-dependent target proteins, as CREB is maximally phosphorylated in HTLV-transformed cells [114]. Compared with primary CD8^+^ T-cells, much higher levels of transcription from the HTLV-1 LTR has been observed in primary CD4^+^ T-cells, suggesting that cellular factors within CD8^+^ T-cells may be insufficient to support efficient functioning of the HTLV-1 promoter [115,116]. 

Although BLV may be present in monocytes, granulocytes, CD4^+^ T cells, CD8^+^ T cells and γ/δ T cells, the major cellular target of BLV is the B lymphocyte [117,118,119,120,121]. As discussed above for HTLV, CREB/ATF complexes present in BLV-infected lymphocytes activate LTR-directed gene expression in the presence of PKA or Ca2+/calmodulin-dependent protein kinase IV, and are the major transcription factors involved in the early stages of viral expression [95]. In addition, the U3 region of the BLV LTR includes B-lymphoid-specific *cis*-regulatory elements for two Ets members PU.1 and Spi-B proteins [100]. During normal development, the tissue distribution of PU.1 is restricted to cells of the hematopoietic lineage, including B cells, megakaryocytes, granulocytes, mast cells, immature erythrocytes, and myeloid cells [122,123,124]. In contrast to PU.1, Spi-B expression is limited to B cells and immature T cells [125,126]. These findings suggest that PU.1 and Spi-B protein play a role in the B-cell tropism of the virus and could be involved in the early stage of viral replication.

HTLV and BLV gene expression in monocytes and DCs is mediated primarily through the enhanced DNA-binding activity of AP-1 to TRE-I. In HTLV-1 infected cells of the monocyte/macrophage lineage, stimuli that trigger monocytic differentiation or activate protein kinase C (PKC) pathway enhance the expression and DNA-binding activity of AP-1 transcription factor (Fos/Jun) [34,127,128]. Monocytic differentiation-induced activation of PKC may activate AP-1 expression resulting in up-regulation of basal LTR activity [129]. It remains possible that AP-1 proteins may function to activate the HTLV-1 LTR in the absence or presence of existing ATF/CREB proteins through the formation of heterodimers.

BLV has also been detected in other tissues including endothelial and mammary epithelial cells [130,131,132]. However, no enhancers contained within LTR that would be associated with these tissues-specific expression of the virus were described. The ability of the LTR to direct mammary tissue-specific transcription is known for MMTV [133]. In contrast to BLV, mammary gland enhancer (MGE) has been mapped to the 5′ LTR of the U3 region of MMTV (Figure 1) [134]. A number of factors have been identified that bind to this enhancer region, including MP4, MP5, AP-2, CTF/NF1, MAF and MGF (Stat5a) [135,136,137]. Many of these factors are developmentally regulated in the mammary gland. DNA binding of MAF, MGF and MP4 are all activated by prolactin, epidermal growth factor or transforming growth factor (TGF) α [138]. The relationship between infection of the bovine mammary epithelium by BLV and these factors has not been explored yet.

## 5. Transactivation: Activation of LTRs by Virus-Encoded Proteins

Basal transcription from the LTR is insufficient to promote productive virus replication. Delta- and Lenti-retroviruses are able to increase the rate of gene expression through virus-encoded transcriptional activator proteins (transactivators). Although the effect is very similar in BLV/HTLV-1/HIV, the mechanisms are quite different [15,139,140]. Activation may occur by interaction with DNA sequences in U3 (e.g., BLV and HTLV-1 Tax proteins) or by binding to a specific sequence near the end of a 5′ TAR RNA transcript (e.g., HIV-1 Tat protein, reviewed elsewhere in [141,142]). HTLV-1 and BLV Tax proteins further transactivate a series of cellular genes in lymphocytes (transcription factors, cell cycle regulators, protein kinases and phosphatases), which are predicted to modulate apoptosis, proliferation, promote immortalization and ultimately lead to oncogenesis. In particular, Tax mediates the activation of gene expression via the NF-κB pathway. Tax decreases the stability of various inhibitors of NF-κB in the cytoplasm (such as IκBα and WW domain–containing oxidoreductase) and induces NF-κB nuclear translocation [143]. In nuclear foci, Tax interacts with the RelA subunit of the NF-κB complex when bound to κB sites within the LTR, increasing activation of gene expression [143,144]. 

Deltaretroviruses induce a polyclonal expansion of lymphocytes that evolves into an aggressive monoclonal leukemia in approximately 5% of infected individuals after a variable but frequently long period of latency [145,146]. Although Tax and HBZ are major determinants, additional events are necessary for a particular cellular clone to progress into the tumorigenic state. Although Tax expression provides a proliferative advantage, expression of viral proteins exposes the infected clone to host immunity. Tax is clearly essential during the initial stages of infection, but may not be required later on during the transformation process. This is exemplified by the generation of clones that contain proviruses with 5′ deletions unable to express Tax [147]. 

Sense HTLV-1 proviral expression can also be inhibited by the antisense protein HBZ, which counteracts Tax by competing for binding to CREB and CBP/p300 [9] and other ATF/CREB factors like CREM and ATF-1 [148]. HBZ can hamper the formation of an active transcriptional complex on the viral promoter by specifically targeting these cellular factors. 

Since Tax is an immunodominant target antigen for cytotoxic T-lymphocytes (CTLs) in vivo, the proviral clones might escape from the host immune system by suppressing Tax expression. Therefore, immortalized cells that induce weaker CTL responses are favored during the development of adult T-cell leukemia (ATL) [149]. During ATL development, escape from CTL recognition results from spontaneous genetic changes (nonsense mutations, deletions or insertions) in proviral sequences encompassing structural genes, the 5′ LTR sequence and the *tax* gene (in particular, mutations in Tax CTL epitopes) [149,150,151]. Consequently, most nonsense mutations result in a non-functional Tax protein. Premature stop codons present in the *tax* gene at positions 7464, 7469, 8045 were identified in HTLV-1 carriers and patients with ATL. Multiple deletions and/or insertions in the *tax* gene in ATL patients is a mechanism that abrogates transactivation activity and promotes escape from the immune system [151], in particular from CTLs [52,149]. Moreover, some defective proviruses lacking the 5′-LTR due to partial deletion consequently loose transcriptional regulation by Tax. Defective proviruses are rare at the beginning of infection, but later on clones with defective-type proviruses preferentially expand and become predominant [152].

The U3 region of the BLV LTR also contains three TREs (TRE1, TRE2, and TRE3), which are important for Tax-mediated activation of the LTR. A NF-κB-binding site is located between TRE2 and TRE3 of the U3 region [153]. This site is associated with strong activation of BLV transcription in the presence of NF-κB and Tax. Since Tax expression is usually undetectable during viral latency in infected B-lymphocytes, NF-κB protein activity may provide the initial trigger for BLV transcription. This basal transcription may lead to synthesis of Tax, which together with NF-κB synergistically activates BLV transcription [98]. 

As in HTLV-1, mutations that affect *tax* gene expression also occur in BLV. Naturally occurring single amino acid substitution E303K impairs Tax function and induces the silent BLV phenotype in BLV-induced B-cell tumor [58]. Reverse genetics demonstrated that the defect could be compensated with a functional Tax gene [154]. The D247G mutation in Tax enhances viral expression and propagation in vitro but fails to generate a higher proviral load and expression of viral RNA in vivo in comparison with wild-type BLV [155]. Amino acid substitutions between residues 240 and 265 of Tax results in a significant increase of LTR transactivation [156]. 

In BLV-infected cattle, different Tax variants can be associated with changes in white blood cell numbers [157]. A major difference between the aleukemic, persistently lymphocytotic and lymphosarcoma stages correlated with the E51G substitution located in the putative zinc finger domain of Tax. Mutations in the Tax zinc finger destroys transactivation activity but maintained its transforming capacity in vitro [158]. BLV Tax L233P substitution in infected cattle is also recognized to prolong the incubation period for developing tumors [159].

Collectively, these data reveal that Tax is an essential regulator of viral transcription upon interaction with the TRE elements and CREB/ATF transcription factors. Tax nevertheless becomes counter-selected by a series of mechanisms including proviral mutations. 

## 6. Transcriptional Repressors

LTRs also interact with repressors of transcription (Box 1). These include three main groups of cellular transcription factors: C2H2-type zinc finger proteins (e.g., Krüppel-associated-box KRAB), Specificity proteins (e.g., SP1) and Basic helix-loop-helix-zipper proteins (e.g., AP4). These repressors are predicted to induce and/or maintain retroviral latency. These negative regulatory proteins may use epigenetic mechanisms for suppression of viral gene expression. For example, AP-4 and E47 recruit chromatin-remodeling complexes to specific promoter elements and induce local changes in chromatin conformation of the LTR [80,97,102]. After proviral integration into the host cell chromatin, retroviral genomes can be reversibly silenced through multiple interconnected mechanisms, establishing a state of post-integration latency. Although different posttranscriptional mechanisms participate in maintaining the latent state, it is usually a blockade at the transcriptional level that gives a major contribution to the establishment of latency.

Box 1 **Key transcription factors associated with retroviral latency.**

**C2H2-type zinc finger proteins (ZNFs)** represent large family of the transcription factors characterized by multiple tandemly repeated zinc fingers, which allow high flexibility in target recognition [160]. Most of them contain Krüppel-associated-box (KRAB) repression domains, which induce repression of transcription in conjunction with TRIM28 (tripartite motif-containing protein 28, also known as KRAB-associated protein 1, KAP1) [161]. The biological significance of several hundred of the KRAB-ZFPs has been already determined; however, in most cases it is still unknown [162]. Interestingly, the KRAB-ZFPs have the potential to regulate retroviral gene expression. One of them, named ZNF10 was found to recognize the NF-κB and Sp1 binding sites of HIV-1 LTR, and in conjunction with TRIM28, H3K9 methyltransferase ESET (SETDB1), HP1-gamma and NuRD histone deacetylase (HDAC) complex, suppress LTR-mediated transcription [163]. In addition, other factors of this family such as ZNF350 (ZBRK1) and ZNF1, are able to repress HIV-1 replication through binding the −145 to −126 region of the HIV-1 LTR in conjunction with TRIM28 and HDAC2, consequently suppressing gene expression [164]. Interestingly, a new zinc finger protein ZFP809 in stem cells that directly recognizes proviral DNA of MLV and recruits TRIM28 to mediate chromatin modification and transcriptional gene silencing, which prevents further viral spread [162]. In addition, novel Krüppel type-zinc finger protein, HUB1 (HTLV-1 U5RE binding protein 1) is a potential repressor of the LTR-mediated viral gene expression for HTLV-1 [165]. HUB1 has a KRAB-like domain recognizing the TCCACCCC sequence within U5 repressive element (U5RE) of the HTLV-1 LTR, which is also the same core motif for the Sp1, Sp3 transcription factors [165]. However, to fully understand the mechanism of HUB1’s repressive effect on HTLV-1 gene expression, molecular characterization of protein domains within HUB1 need to be determined [166]. Collectively, these results suggest that KRAB-ZFPs suppress retrovirus LTR-driven gene expression and may have novel antiviral therapeutic potential [162]. One main question that remains yet to be answered is why retroviruses (especially those as highly variable as HIV-1) have not mutated to avoid KRAB-ZFPs repressor proteins. A possible answer to this central question is that transcriptional repression through KRAB-ZFPs remains advantageous for retroviruses to establish a latent state.

    Another C2H2 zinc finger transcription factor, COUP-TF-interacting protein 2 (CTIP2), was found to repress HIV-1 transcription via a direct association with the LTR-bound transcription factor Sp1 and chicken ovalbumin upstream promoter transcription factor (COUP-TF).  CTIP2 also facilitated HIV Tat protein relocation to transcriptionally inactive regions of chromatin [167]. CTIP2 recruits histone deacetylase (HDAC) 1, HDAC2 and histone methyltransferase SUV39H1 enzymes to the viral promoter, which promote local histone H3 deacetylation and increases local histone H3 lysine 9 methylation, respectively [168]. This allows heterochromatin protein 1 (HP1) binding to the viral promoter and inducing formation of heterochromatin, leading to HIV-1 silencing [169]. The potential role of CTIP2 in transcriptional regulation of the HTLV-1 promoter has also been demonstrated. Chromatin immunoprecipitation (ChIP) assay revealed the possibility of recruitment of CTIP2 to the HTLV-1 LTR in a latently infected cell line (TLom1). In addition, the post-translational modification of CTIP2 impaired the ability of CTIP2 to repress Tax-mediated HTLV-1 transactivation [170]. This observed repression of HTLV-1 gene transcription may be exerted via CTIP2 binding to Sp1; however, this mechanism needs to be elucidated. CTIP2 is key factor in promoting the formation of heterochromatin and may play crucial role in establishing latency by preventing viral reactivation [170]. Constitutive presence of this factor in microglia cells may explain mechanisms underlying establishment and maintenance of HTLV-1 latency in the central nervous system.

**Specificity protein (Sp)** is a well-known family of transcription factors that includes Sp1, Sp2, Sp3 and Sp4, which are implicated in cell growth, differentiation, apoptosis and carcinogenicity [171]. Sp1 promotes HIV-1 gene transcription by direct binding to the three GC boxes adjacent to the TATAA sequence. Sp1 can also serve as an anchor for indirect binding of such transcription factors as COUP-TF, NF-IL-6, NF-κB, positive transcription elongation factor b (P-TEFb) and Tat proteins for optimal HIV-1 enhancer activation. In comparison with transcriptional activators Sp1 and Sp4, Sp3 protein represses promoter activity. In microglial cells, which serve as a latent reservoir of HIV-1, Sp1 and Sp3 proteins act as antagonists on LTR-directed transcription, where Sp3 is able to repress positive action of the Sp1, as well as the COUP-TF factor. This regulatory mechanism of Sp3 involves competition with Sp1 for occupancy of the GC boxes. Over-expression of the Sp3 in microglial cells is also able to inhibit Tat-mediated HIV transactivation [171].

    In the HTLV-1 LTR region six Sp1 binding sites were identified: two Sp1 binding sites located in U3 5′-LTR region that are crucial for basal and Tax-transactivated promoter expression, two Sp1 binding sites located in U5RE sequence of LTR region that acts as repressor for HTLV-1 gene expression in absence of Tax, and two new functional Sp1 sites located within the R region that acts as negative *cis*-regulatory elements [165]. However, for the recently discovered Sp1 binding sites within R region, a precise mechanism of repression of LTR-driven transcription need to be further studied. One possibility is that Sp3 competes with Sp1 for the same DNA binding site or from steric hindrance between repressors and positively acting transcription factors [91,165,172].

**Basic helix-loop-helix-zipper (bHLH-ZIP)** family includes transcription factors Activating enhancer binding protein 4 (AP-4) and E protein (E47). They contain a basic domain, responsible for DNA binding on the E-box motif (CAGCTG), and HLH and ZIP domains, which are implicated in protein oligomerization [173]. For the regulation of HIV-1 transcription, AP-4 binds to the 3′ E-box element (CAGCTG) located between the TATA box and initiator element (−21 to −16) of the U3 core promoter in HIV-1 [174]. Over expression of AP-4 in HEK 293T cells mediate repression of HIV-1 transcription through two mechanisms: (1) AP-4 bound to the 3′ E-box can block binding of TATA binding protein (TBP) to TATA box in LTR promoter, and (2) AP-4 can mediate the recruitment of HDAC1 to the LTR in vivo [175,176,177].

    In addition, another bHLH factor, named E47 has shown silencing activity of the HTLV-1 promoter, both in the presence and absence of Tax. It is possible that E47 binds to the putative E box in the promoter 28 bp upstream of the TATA box and negatively modulates the binding of HAT p300/CBP to the CRE-binding protein site within 21-bp Tax-responsive elements [176,178]. The close proximity of these E boxes to the TATA region appears to be specific to the LTRs of HIV-1 and HTLV-1 [176]. Interestingly, the overlapping of E box elements and the cyclic AMP responsive elements (CREs) in the BLV LTR has also been proposed as a strategy to allow better silencing of viral transcription. Notably, binding to the overlapping CRE and E box elements within the BLV TREs is mutually exclusive [80]. In addition, transcription factors binding to the E box motifs may negatively modulate the binding of CREB/ATF complexes to the overlapping CREs and further to their transcriptional coactivators, possessing acetyltransferase activities, thus leading to the repression of BLV transcription [80]. A potential role for AP-4 transcription factors in binding to the three E box motifs within BLV LTR and overlapping the CREs has been described [97]. However, direct binding of this factor has not been reported yet, and the identification of the adequate factors binding to the E boxes of BLV LTR region still remain to be established.

## 7. Regulation of LTR Transcription through Hormonal Signaling

Modulation of virus replication by hormones is physiologically relevant, and crucial to our understanding of sex-differences in virulence and immunity to retroviruses [179,180]. Many retroviruses have regulatory elements responsive to hormonal transactivating factors. An example of this is estrogen receptor-1 (ESR-1), which plays an important role in suppression HIV transcription and is required to maintain HIV-1 latency [181]. The accumulation of ESR-1 at the LTR rises in the presence of β-estradiol. ESR-1 together with β-catenin associate as corepressor complex for the HIV-1 LTR. It is unclear if there is an ESR-1 binding site within the LTR, suggesting that the recruitment may be indirect. A potential mediator of ESR-1 recruitment is Sp1 which binds to three sites in the HIV-1 core promoter on the LTR, at position −143 nt from the transcription initiation site [182,183]. These studies demonstrate that in HIV-1-infected cells, estradiol represses HIV-1 replication by directly altering HIV-1 transcriptional activation. These observations are in agreement with the negative regulation of HIV-1 replication observed in infected PBMCs when treated either with 17-β-estradiol (E_2_) or progesterone (P_4_) [184].

The current knowledge of the endocrine system control of HTLV-1 viral gene expression is very limited [185]. No hormone receptors have been identified in the HTLV-1 LTR. While daily prednisolone slows progression of HAM/TSP [186], the correlation with Tax is unknown. HTLV-1 Tax affects expression of estrogen-responsive genes to enhance breast epithelial cell replication, through estrogen (E2) receptor α (ERα) and classical NF-κB pathways [187,188]. On the other hand, there is still no clear epidemiological evidence that correlates HTLV-1 infection and breast cancer. Further studies on hormonal signals, which have a potential to regulate HTLV transcription and viral production would be valuable to understand the pathophysiology of this infection.

In contrast, BLV infection has been correlated with breast cancer development, although these observations are still controversial [189,190,191,192]. A glucocorticoid-responsive element (GRE) is present in the U3 region of BLV LTR [193]. A mutation in this site decreases basal LTR activity as evidenced by a reporter-based in vitro assay. Moreover, the GRE element increases LTR transcription in response to dexamethasone in the presence of Tax [99]. In this regard, the hormone responsiveness of BLV may have similarities to that described for MMTV [194]. Similar to what is observed in MMTV infected mice, BLV infected cattle showed an increase in estrogen and progesterone levels during pregnancy, followed by a sharp increase in glucocorticoid levels toward the end of term and an increase in BLV antibody levels within a month after parturition [195,196,197]. These observations suggest that steroid hormones might be involved in the induction of viral expression in BLV.

Immunocytochemical studies of mammary epithelial cells infected in vivo with BLV suggest that these cells are in a state of latency and after administration of glucocorticoids and prolactin together, may stimulate the production of viral particles [99]. However, whether one GRE binding site may be a critical determinant of tissue specificity and pathogenicity in infected animals remains yet to be explored (Figure 1). In addition, further studies are necessary to determine if BLV is able to escape the immune response by maintaining a long-term latency in mammary cells until late pregnancy, prior to an increase in the level of glucocorticoids during lactation which induces viral replication and favors the transmission to the newborn.

Repression of viral expression is a major strategy to evade developed by Deltaretroviruses immune response. Considering that (i) several stressor factors are associated with animal handling in production facilities, (ii) animals exposed to stress stimuli present higher levels of glucocorticoids and (iii) the role of glucocorticoids as strong immunosuppressive agent has been extensively demonstrated, it would be possible under certain physiological circumstances (e.g., immunosuppressive state) that glucocorticoids might favor BLV replication and spread in vivo. Opposite to what was observed from in vitro studies, mutations in the GRE region of BLV did not affect virus infectivity and replication using an in vivo ovine model of BLV infection [94]. In agreement with this report, administration of dexamethasone to chronically infected cattle does not increase of viral replication [198]. Further studies are necessary to understand which is the role of glucocorticoids in BLV infection and the implications of virus reactivation on BLV pathogenesis.

## 8. Impact of Naturally Occurring Functional Mutations in LTRs

Retroviruses, similar to other RNA viruses, exhibit generally a high mutation rate because of polymerization errors occurring during DNA synthesis by reverse transcriptase (RT), which lacks proofreading activity. Cytidine deamination of RT products through APOBEC3G activity also contributes towards hypermutation of proviral DNA [199]. Provirus variation has important implications for virus replication, immune escape, and disease progression. Escape mutations in the HIV envelope protein (Env) contributes towards immune evasion within a HIV-1 infected individual [200,201]. Indeed, the presence of tissue-adapted variants plays an important role in the pathogenesis of HIV disease. For any given human or animal retrovirus, there is evidence that changes in host cell tropism and modulation of the viral pathogenic properties can be determined by single point mutation variation in the LTR [202,203,204]. Specific examples of sequence variation in the LTRs of select retroviruses are presented in Table 1.

LTR sequence alteration can affect the modulation of viral expression and latency [205,206]. Unlike other retroviruses, HTLV and BLV show a low degree of genetic variation. *In vitro*, the BLV RT is 10-fold more faithful than other retroviral polymerases. The error rate in BLV is 4.8 × 10^−6^ and in HTLV-1 is 7 × 10^−6^ nucleotides, as compared to 2.5–5.9 × 10^−4^ for purified HIV-1 RT and 3.4 × 10^−5^ measured during single-cycle HIV-1 infection [207,208,209,210,211]. The relatively lower diversity of HTLV and BLV may also reflect the fact that replication of these viruses is primarily by clonal or mitotic expansion, rather than by reverse transcription and virus replication [212,213]. Alternatively, mechanisms of transmission between animals may be a bottleneck that does not allow large modifications in the genome. Despite the low genetic variability, it is believed that small variations in the deltaretrovirus LTR region may modify the binding ability of transcription factors and could influence gene expression [153]. 

Although most of the deltaretroviral genome remains stable, variations are noted in the promoter region of these retroviruses. Among these, a G(232)A transition in the CREB-binding site in the TRE-1 of the HTLV-1 LTR is commonly present in HTLV-1 asymptomatic carriers. This nucleotide variation correlates with increased viral proliferation and proviral load [214]. Furthermore, a provirus derived from a HAM/TSP-affected individual harboring a G(174)A change in the TRE-II showed a two-fold lower transcriptional activation level compared to wild-type in vitro [215]. Another example is the ATF-2 binding site within the R region (+202− +246) that is associated with transcriptional down-regulation of the integrated HTLV-1 LTR promoter [203]. Naturally occurring mutations in the HTLV-1 LTR R region affecting ATF2 binding have been identified particularly among HAM/TSP patients [203]. A series of mutations (at positions +220, +223 and +225) result in diminished ATF2 binding and de-repression of the LTR promoter in transient transfection assays [203]. 

In BLV, few studies have evaluated proviral LTR sequences for the presence of single nucleotide polymorphisms (SNP), insertions and deletions [59,153,216]. SNPs in the U3 region occur both within and outside of transcription factor binding sites and promoter elements. The most frequent changes observed in regulatory elements of the U3 region of the LTR are characterized by substitution of G(−133)A/C in the TRE-2, substitution T(−65)C in the GRE and substitution of T(−41)A, T(−37)A and T(−36)C in the TATA Box. The remaining variations (substitution of A(+150)G, T(+161)C, TC(+188/9)CT and T(+ 190)C) are identified in the DAS regulatory element in the R region. Despite some variability within the U5 region, there is no accumulation of specific mutations within well-defined regulatory elements. Further analysis reveals the presence of insertions and deletions: four deletions at position C(−72)del of GRE, T(−11)del of CAP site, TC(+ 188/9)del of BoxC and A(+ 320)del in a non-regulatory site [153,217]. 

The U3R region of the BLV 5′ LTR is involved in the control of both basal and Tax-dependent transcription of the BLV genome though its interaction with several cellular transcription factors. Therefore, mutations affecting promoter nucleotide sequence might drive potentials differences in the biological properties of diverse BLV strains. In support of this, the spontaneous substitution T175C within the TATA box is associated with virus productivity in vitro, which was closely related with BLV transmissibility [218,219]. 

The functionality of these mutations can be investigated by reverse genetic experiments. In this context, it is noteworthy that high level of promoter activity does not necessarily correlate with efficient replication. Indeed, the substitution of the suboptimal CRE elements in the 3 TREs increases promoter activity but reduces proviral loads [94]. This surprising observation suggests that small variations in the LTRs are required to limit viral expression which is detrimental to efficient replication in presence of an efficient immune response. 

## 9. Role of Non-Coding RNAs in Regulation of Deltaretrovirus Transcription

Non coding RNAs of cellular or viral origin can modulate LTR-directed transcription, replication and pathogenesis [220]. 

### 9.1. Host lncRNAs 

Long non-coding RNAs (lncRNAs) are defined as RNA transcripts larger than 200 bp without any coding potential [221]. They appear in the genome in form of antisense lncRNAs, intronic lncRNAs and intergenic lncRNAs. A main function of lncRNA is to regulate gene activity through interactions with chromatin, especially to suppress gene expression [222]. 

In HIV, noncoding repressor of NFAT (NRON) which is a highly expressed lncRNA in resting CD4^+^T lymphocytes can act as a negative regulator of transcription factor NFAT. Since NFAT promotes HIV-1 expression by binding to the downstream sequence of TAR, NRON is able to suppress HIV transcription [223]. NRON also suppresses viral transcription by degradation of HIV Tat. NRON directly links Tat to CUL4B and PSMD11 (ubiquitin/proteasome processing proteins), facilitating Tat degradation, thereby contributing to HIV-1 latency [224]. Another example of regulation HIV-1 transcription by lncRNA is by HIV-1 enhanced lncRNA (HEAL), which recruits the fused in sarcoma (FUS) RNA-binding protein to the HIV-1 LTR resulting in the recruitment of the HAT p300, thus increasing the transcription of HIV-1 [225]. lncRNA metastasis-associated lung adenocarcinoma transcript 1 (MALAT1) increases HIV-1 transcription by reduction in the epigenetic silencing of HIV-1. MALAT1 can limit the amount of catalytic subunit EZH2 from the PRC2 complex and loss of PRC2-mediated H3K27me3 at the LTR promoter [226]. 

In HTLV-1, the lncRNA antisense noncoding RNA in the INK4 locus (ANRIL) cooperates with EZH2 to enhance the proliferation of HTLV-1 infected cells [227].

### 9.2. Viral lncRNAs

In HIV, antisense transcripts of ncRNAs suppress HIV gene expression by recruitment of a repressive complex containing DNMT3a, HDAC-1 and EZH2 [228,229,230]. These antisense transcripts are detected mainly in active CD4^+^ T cells, while being almost undetectable in resting CD4^+^ T cells [231,232]. 

HTLV-1 and BLV also transcribe antisense RNAs, respectively HBZ and AS1/2 [10,233]. Although poorly translated, the HTLV-1 HBZ gene expresses a polypeptide that inhibits Tax activity (see discussion above) [18,233]. There are different spliced and polyadenylated isoforms of HBZ RNA whose abundance varies from cell to cell. Significant amounts of HBZ RNA are present in the nucleus of infected cells isolated from patients. *HBZ* RNA is expressed mainly in ATL cells, and primarily localizes to the nucleus, where it functions as an lncRNA to induce T cell proliferation [233,234,235]. However, it remains to be elucidated how *HBZ* RNA functions to transforms T cells [147]. 

BLV provirus also constitutively expresses antisense transcripts in all leukemic and asymptomatic samples. Similarly to the *HBZ* RNA of HTLV-1, the transcripts are retained in the nucleus, indicating that the main functions of AS1-S/L and AS2 are exerted by their lncRNA form. These antisense RNAs may play role in regulation of BLV, silencing of the 5′-LTR and/or leukemogenesis [10].

### 9.3. Host miRNAs

Several host microRNAs (miRNAs) were demonstrated to have direct or indirect effects on retroviral replication. In HIV-1, a series of miRNAs (miR-28, miR-125b, miR-150, miR-223 and miR-382) are enriched in resting CD4^+^ T cells as compared to activated CD4^+^ T cells, interact directly with the 3′ ends of HIV-1 mRNA and decrease replication [236]. The mir-198 is an example of a host miRNA that indirectly affects HIV-1 transcription by targeting the 3′ untranslated region (3′UTR) of cyclin T1 mRNA [237]. As Cyclin T1 is required for Tat transactivation of the HIV-1 LTR, miR-198 functions to restrict HIV-1 replication in monocytes through repression of cyclin T1 expression, which results in suppressing viral LTR activation [237]. 

HTLV-1 Tax downregulates host miRNAs to promote continuous viral transcription [238]. As discussed earlier in this review, transcription of the HTLV-1 viral genome is largely carried out by the complex formation of Tax/CREB with the CBP/p300 HAT. Recruitment of CBP/HAT p300 acetylates the histones of proximal nucleosomes and promotes chromatin remodeling which results in an euchromatic state. Tax downregulates both miR-149 and miR-873, which target HAT p300 associated with chromatin remodeling factors in T cells. Tax also interacts with and inhibits Drosha, thereby affecting the cell miRNA transcriptome [238,239]. 

### 9.4. Viral miRNAs 

Viruses also express miRNAs that control the viral life cycle, cell survival and immune evasion. Many viral miRNAs downregulate the expression of host factors harboring antiviral activity [240]. In HIV, the *pol*-derived miR-H3 targets the 5′-LTR TATA box and activates transcription of the viral promoter [241,242]. In contrast, HIV-1 produces large amounts of TAR-encoded miRNA-like small RNA (miR-TAR-3p and miR-TAR-5p) from a non-processed transcript that inhibits HIV-1 LTR activity and induce chromatin remodeling. Increased levels of TAR-derived ncRNAs result in recruitment of HDAC and methyltransferases involved in local epigenetic modification [243]. 

BLV transcribes a cluster of viral miRNA through RNA Pol III dependent promoters. BLV miRNAs are abundantly expressed in both bovine B-cell tumors and in experimentally-infected bovine calves [244]. The miRNAs can interfere with host immunity and are associated with reduced expression of genes associated with B-cell function and differentiation, specifically IGJ, PAX5, BLIMP1, and BCL6 [244]. Ablation of BLV miRNAs impacts fitness and suppression of oncogenicity in the natural host [245]. The main mode of action the BLV miRNAs occurs through increased proliferation of B lymphocytes [246]. In contrast to BLV and HIV, no virally-encoded miRNA could be identified in HTLV-1. 

The existence of HTLV-1 and BLV antisense transcripts implies that the LTR promoter is bidirectional. The abundance of sense and antisense transcripts fluctuates in transient bursts via a still unexplained mechanism [74,247]. During the chronic stage of infection, 5′-LTR driven transcription is repressed while antisense RNAs are present [10,248]. This dual regulation can be explained by epigenetic mechanisms (histone modifications and site-specific DNA methylation) that prevent transcription factor binding at the 5′-LTR [49,52,54,249]. The consistent expression of non-coding RNAs in tumor cells suggests that they play a key role in BLV regulation and pathogenesis. 

## 10. Conclusions

Retroviruses having a DNA intermediate in their replication cycle can undergo transcriptional silencing and hide from host immunity. Understanding the molecular mechanisms underlying silencing are necessary to clear the viral reservoir from an infected host (Table 2).

**Figure 2:**
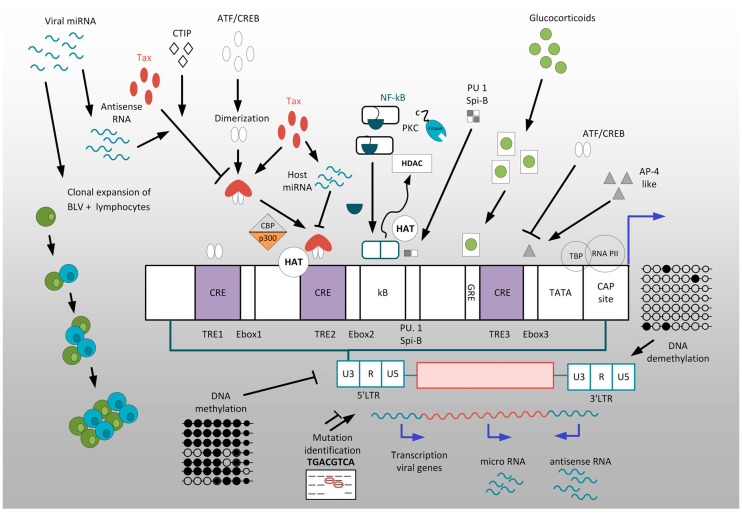
A schematic diagram of regulation of expression in BLV. The scheme illustrates the interactions that induce 5′LTR transcription: TRE element and the Tax/ATF/CREB and CBP/p300 complexes; NF-kB, PKC, and HAT; glucocorticoid receptor (GR), glucocorticoids and glucocorticoid response element (GRE); demethylation within 3′-LTR; some mutations within regulatory elements of the LTR; and the interaction that repress 5′LTR transcription: antisense RNA and Tax; CTIP and Tax; NF-kB and HDAC; AP-4 like protein ATF/CREB and E box; methylation within 5′-LTR; some mutations within the LTR. Black sharp arrows indicate an induction of 5′LTR transcription, black blunt arrows indicate repression of 5′LTR transcription. Clonal expansion of BLV^+^ lymphocytes is another mode of viral propagation. Transactivation of transcription at the viral LTR is a critical role of Tax in BLV infected cells as this ultimately leads to the expression of all viral genes. The LTR is divided into the U3, R and U5 regions. The U3 region is of significant importance in Tax-mediated transcription and thus is highlighted in the expanded box. This region contains three TRE-1,2, 3 and CRE regions; Ebox1,2,3; κB; PU.1/Spi-B region; GRE; TATA box and CAP site.

Transcriptional and posttranscriptional mechanisms participate in initiation and/or maintenance of latency. In this review, we outlined a wide range of mechanisms underlying deltaretrovirus transcriptional regulation and latency (Figure 2).

Activation of LTR-driven sense transcription may have clinical outcomes due to its therapeutic potential in a shock-and-kill strategies. This clinical approach consists of three-steps: (1) the reactivation of viral transcription from latently infected cells, followed by (2) the killing of cells expressing viral antigens, and (3) anti-retroviral therapy for protection of uninfected cells. Transcription can be targeted by HDACs inhibitors like valproic acid (VPA) or methylation inhibitors such as 5-azacytidine (5-AzaC) [49,78]. HDACi used alone can be used to induce expression of BLV and HTLV-1 and reduce proviral loads [37,38,78]. However, the effect is only transient in HAM/TSP patients [40], and may be related to temporal fluctuations in viral RNA and response to immune control mechanisms in these patients [250,251,252]. Improved therapies include VPA combinations with zidovudine (AZT) or cytokines (IFNα) [253,254,255]. 

These therapeutic approaches are not practical for BLV-infected cattle in agricultural settings, mainly due to animal management restrictions and economic costs. However, BLV infection in cattle could be used as an in vivo model for HTLV viral infection in humans. For BLV, applying test-and-elimination strategy led to the successful eradication of the EBL in some European countries. Since this strategy is economically untenable in countries with high levels of BLV prevalence, test-and-segregation programs have been recommended but are rarely implemented. Therefore, a better understanding of modes and mechanisms of transmission of BLV, might aid the development of novel control strategies for BLV. We hypothesize that external stimuli (e.g., stressor factors associated with the environment and animal handling in production, etc.) might trigger virus reactivation from latency, contributing to higher viral loads and virus transmission. In this scenario, reducing stressors as well as separating high provirus load herds could prevent BLV transmission and reduce overall prevalence.

Moving forward, an improved understanding of the fundamental regulatory mechanisms controlling deltaretroviral transcription will assist in the development of novel and more efficient anti-retroviral prophylactic strategies and therapeutic agents.

## Figures and Tables

**Table 1:** Examples of sequence variation in the LTRs of select retroviruses and their impact on phenotypic change and clinical outcomes.

Genus	Species/Strain	The Type of Mutation within LTR	Phenotypic Change and/or Clinical Impact
Alpharetrovirus	endogenous avian retrovirus	lack the CCAAT/enhancer elements, contain only one CArG box and Y box	transcriptionally inactive
	rous sarcoma virus	5-bp deletion in the a3 site, which removes most of the CCAAT/enhancer element	abolishes binding of the vitellogenin gene-binding protein (VBP) bZlP factor to this site
	avian leukosis virus of subgroup J	11 bp deletion in transcriptional regulatory element ABI REP1 of U3 region	associated with the occurrence of hemangioma
	avian leukosis virus (AF115-4)	recombinant subgroup B avian leukosis virus with a subgroup J	induction of lymphoid leucosis (LL) and not the expected myeloid leucosis (ML)
Betaretrovirus	mouse mammary tumor virus (Mtv-17)	single G to A transition at position-75 in the binding site for nuclear factor 1 (NF-1)	transcriptionally defective, decreased hormone-induced transcription from promoter as well as NF-1 binding in vitro
	mouse mammary tumor virus (from B6 EL4 tumor)	deletion of 491 bp (approximately −655 to −165)	elevated transcriptional activity in brain, heart, and skeletal muscle-in the absence of hormone
	mouse mammary tumor virus	deletion of sequences between −201 and −344	inappropriate expression in heart, brain, and skeletal muscle
Gammaretrovirus	feline leukemia virus	repeats of 40 to 74 bp in the upstream region of the enhancer (URE)	repetitive URE conferred an enhancer function upon gene expression in myeloid cells, suggesting its association with tumorigenic potential in myeloid cells
	feline leukemia virus	21-bp tandem triplication beginning 25 bp downstream of the enhancer	triplication-containing LTR acts preferentially in a cell-type-specific manner; induction of tumors of a particular phenotype
Deltaretrovirus	HTLV-1	A to G at position +223 R region of LTR	no RF complex is detected; transcriptional de-repression LTR promoter
Lentiretrovirus	human immunodeficiency virus type 1 (CRF01_AE strain)	1-nucleotide deletion in upstream NF-kappaB site of the tandem enhancer motif	conversion NF-κB into a binding site for GABP transcription factor, results in higher replication rate and transmission efficiency of CRF01_AE compared with subtype B
	human immunodeficiency virus type 1	CCAAT/enhancer-binding proteins (C/EBP) site I (C to T at position 3)	high relative affinity for viral protein R (Vpr), development of the (HIV-1) associated dementia (HIVD)
	human immunodeficiency virus type 1	C-to-T change at position 5 within Sp site III and 5T mutation within Sp site II	altered recruitment of Sp isoforms, correlate with disease progression and severity
	human immunodeficiency virus type 1 (CRF02_AG, CRF22_01A1)	extension of 4-nt AP-1 site to a 7-nt AP-1 motif “TGACACA”	massively promoted latency establishment
	human immunodeficiency virus type 1	NF-kB-proximal Sp element (Sp site III) alterations of the guanine at position four: GGAG to GGAA	LTR sequence variation and adaptation to influence cell type-specific viral replication and gene regulation
	human immunodeficiency virus type 1	5′-TCTAA-3′ variant TATA box	impair TFIID-directed transcription, severely impaired Tat-dependent transcription
	human immunodeficiency virus type 1	polymorphisms: G to A at position +26 and G to A at position +32 of the loop and bulge of TAR sequence	impaired Tat responsiveness, low-level gene expression

**Table 2:** Summary of mechanisms underlying transcriptional regulation and latency of deltaretroviruses.

No.	Regulatory mechanism
1	Epigenetic mechanisms
2	Transcription factors acting as activators
3	Tissue-specific expression of the proviral genome
4	Viral transactivator Tax is critical for viral infectivity and pathogenesis
5	Transcription factors acting as repressors
6	Glucocorticoid hormones can modulate the induction of BLV expression and pathology
7	LTR sequence variation plays an important role in retroviral replication and modulation of viral latency
8	Host and viral ncRNA play important role in regulation of retrovirus transcription
